# Epidemiological characteristics of bacterial lower respiratory tract infections in Jingzhou, Central China, 2020-2024

**DOI:** 10.3389/fcimb.2026.1869915

**Published:** 2026-08-26

**Authors:** Yan Yang, Zihan Wen, Yue Qu, Yao Lei, Quan Tang, Hanyu Chen, Shun Liu

**Affiliations:** 1 Department of Laboratory Medicine, Jingzhou Hospital Affiliated to Yangtze University, Jingzhou, Hubei, China; 2 Department of Scientific Research, Jingzhou Hospital Affiliated to Yangtze University, Jingzhou, Hubei, China

**Keywords:** Central China, COVID-19, epidemiological characteristics, loop-mediated isothermal amplification, lower respiratory tract infections

## Abstract

**Background:**

Lower respiratory tract infections (LRTIs) represent a significant global public health issue and impose a substantial disease burden. However, regional epidemiological surveillance data on bacterial LRTIs in central China remain insufficient. This study aimed to systematically analyze the epidemiological characteristics of patients with bacterial LRTIs in Jingzhou, Hubei Province, China.

**Methods:**

This study included 5,442 hospitalized patients diagnosed with LRTIs between January 2020 and December 2024. Detection of respiratory pathogens was performed both using a chip-based loop-mediated isothermal amplification (LAMP) assay and the standard culture method (SCM). The LAMP assay kit simultaneously detects ten predefined bacterial respiratory pathogens, including *Streptococcus pneumoniae* (*S. pneumoniae*), *Staphylococcus aureus* (*S. aureus*), *Klebsiella pneumoniae* (*K. pneumoniae*), *Pseudomonas aeruginosa* (*P. aeruginosa*), *Acinetobacter baumannii* (*A. baumannii*), *Stenotrophomonas maltophilia* (*S. maltophilia*), *Haemophilus influenzae* (*H. influenzae*), *Legionella pneumophila* (*L. pneumophila*), *Mycoplasma pneumoniae* (*M. pneumoniae*), and *Mycobacterium tuberculosis* (*M. tuberculosis*).

**Results:**

Among 5,442 samples, the overall positive rate of LAMP was 31.86%, with single-pathogen infections accounting for 26.17%. *H. influenzae* (8.18%) and *S. pneumoniae* (7.75%) were the most commonly detected pathogens. Dual-pathogen co-infections predominated among polymicrobial infections, with *S. pneumoniae* and *H. influenzae* representing the most frequent pathogen combination. The LAMP assay demonstrated a higher overall positive rate than SCM, with moderate agreement observed between the two methods for most pathogens. The overall pathogen positive rate exhibited seasonal variation, peaking from April to June. *S. pneumoniae* showed a bimodal distribution, with higher positive rate in May-June and November-December annually. During the COVID-19 pandemic era, *S. pneumoniae* and *H. influenzae* were predominant, while the positive rates of *M. pneumoniae* and *M. tuberculosis* notably increased in the post-pandemic era. School-aged children exhibited the highest positive rate, with *M. pneumoniae* as the predominant pathogen. Pathogen composition was more diverse among adults and older adults.

**Conclusion:**

These findings demonstrate significant age-related and temporal variations in the pathogen spectrum of bacterial LRTIs and provide epidemiological evidence to support empirical antibacterial decision-making and population-targeted prevention strategies. In addition, LAMP may serve as a practical molecular diagnostic tool for rapid bacterial pathogen detection, although its clinical utility in primary healthcare settings warrants further investigation.

## 1 Introduction

Lower respiratory tract infections (LRTIs) remain a major global health burden, causing approximately 2.49 million deaths and ranking as the fourth leading cause of death worldwide according to the 2019 Global Burden of Disease (GBD) study (Vos et al., 2020; Kang et al., 2023). To reduce the disease burden associated with LRTIs, the World Health Organization (WHO) and the United Nations Children’s Fund (UNICEF) launched the Global Action Plan for the Prevention and Control of Pneumonia and Diarrhoea (GAPPD) in 2013, aiming to reduce pneumonia mortality among children under five years old to fewer than 3 deaths per 1,000 live births by 2025 (Qazi et al., 2015). Despite multiple global prevention and control initiatives, the burden of LRTIs remains highly variable worldwide, especially disproportionately affecting populations in low- and middle-income countries (Quach et al., 2022; Sirota et al., 2026). Consequently, further researchs are needed to understand the epidemiological characteristics of LRTIs across different regions.

LRTIs are caused by a variety of pathogens, such as bacteria, viruses, fungi, and atypical pathogens. The most common pathogens including *Streptococcus pneumoniae* (*S. pneumoniae*), *Staphylococcus aureus* (*S. aureus*), *Klebsiella pneumoniae* (*K. pneumoniae*), *Pseudomonas aeruginosa* (*P. aeruginosa*), *Acinetobacter baumannii* (*A. baumannii*), *Stenotrophomonas maltophilia* (*S. maltophilia*), *Haemophilus influenzae* (*H. influenzae*), *Legionella pneumophila* (*L. pneumophila*), *Mycoplasma pneumoniae* (*M. pneumoniae*), and *Mycobacterium tuberculosis* (*M. tuberculosis*). Some previous researchs indicated that among bacterial pathogens, *S. pneumoniae* and *H. influenzae* remain the primary causes of severe LRTIs and related mortality (Bender et al., 2024). However, significant variations exist in pathogen spectrum composition across different countries and regions, time periods, and populations (Aliberti et al., 2021). Furthermore, the COVID-19 pandemic substantially reshaped the transmission patterns and epidemiological characteristics of respiratory pathogens globally through widespread implementation of non-pharmaceutical interventions, changed population immunity structures, and altered healthcare-seeking behaviors (Izu et al., 2023; Zhang et al., 2025).

At present, the main methods used for respiratory pathogen detection in China include the traditional standard culture methods (SCM), real-time quantitative polymerase chain reaction (RT-qPCR), and loop-mediated isothermal amplification (LAMP). Among these, LAMP has rapidly developed in recent years as a molecular diagnostic technique characterized by operational simplicity, high specificity and sensitivity, and the capacity for simultaneous detection of multiple pathogens (Jin et al., 2025). Comprehensive epidemiological data on bacterial respiratory pathogens in China remain limited. Existing studies have primarily focused on pediatric populations and have largely emphasized viral detection, with relatively insufficient investigation of bacterial infections and other atypical pathogens, particularly in central China and the Jingzhou region (Li et al., 2022; Lv et al., 2024). In this study, we enrolled 5,442 hospitalized patients with LRTIs between 2020 and 2024 to characterize the regional epidemiological patterns of the targeted respiratory bacterial pathogens and to assess the impact of the COVID-19 pandemic. Furthermore, we evaluated assessed the agreement between LAMP and SCM, to explore the potential clinical utility of LAMP for bacterial pathogen detection in LRTIs.

## 2 Methods

### 2.1 Study participant

The study population consisted of hospitalized patients with acute respiratory infections admitted to Jingzhou Hospital Affiliated to Yangtze University between January 2020 and December 2024. For temporal comparison, the study period was divided into the COVID-19 epidemic era (2020-2022) and the post-epidemic era (2023-2024). LRTIs were diagnosed according to the WHO criteria (Fitzner et al., 2018): an acute respiratory infection with a history of fever (or measured temperature ≥ 38 °C), cough, symptom onset within the previous 10 days, and requiring hospitalization. The exclusion criteria were as follows: (1) patients with non-infectious respiratory diseases, including asthma, allergic rhinitis, and respiratory tract tumors; (2) patients with immunosuppression or immunodeficiency; (3) patients presenting more than 7 days after fever onset; (4) patients with incomplete clinical data.

### 2.2 Population settings

This study included a total of 5,442 patients. The patients were divided into four groups according to age: children group (0–5 years old) n=870, school-age children group (6–17 years old) n=311, adults group (18–59 years old) n=1,821, and older adults group (≥ 60 years old) n=2,440. The study protocol was approved by the Ethics Committee of Jingzhou Hospital Affiliated to Yangtze University (2026-108-01).

### 2.3 Samples collection

Respiratory specimens were collected prior to the initiation of antimicrobial therapy. The specimens included bronchoalveolar lavage fluid (BALF) or sputum, with BALF preferentially collected whenever possible. BALF samples were obtained by experienced respiratory physicians using fiberoptic bronchoscopy. Briefly, after lavage of the affected bronchial segment distal to the lesion under bronchoscopic guidance, 10 mL of the recovered lavage fluid was collected, transferred into sterile sampling tubes (Gongdong Medical Technology Co., Ltd, Zhejiang, China), securely capped, and immediately transported to the laboratory for analysis. For sputum collection, patients were instructed to rinse their mouths with normal saline prior to expectoration. Under the guidance of trained nursing staff, patients were asked to take several deep breaths and expectorate deep tracheal sputum into a sterile wide-mouthed container, which was promptly delivered to the laboratory. For patients unable to expectorate spontaneously, sterile suction catheters were used to obtain respiratory specimens via nasopharyngeal or oropharyngeal aspiration by trained physicians.

### 2.4 Loop-mediated isothermal amplification

All specimens were tested using a commercial respiratory pathogen nucleic acid detection kit (CapitalBiotech Co., Ltd, Beijing, China). The assay is based on LAMP, which enables rapid amplification and real-time detection of target nucleic acids under isothermal conditions. The assay simultaneously detects ten targeted respiratory bacterial pathogens, including *S. pneumoniae*, *S. aureus*, *K. pneumoniae*, *P. aeruginosa*, *A. baumannii*, *S. maltophilia*, *H. influenzae*, *L. pneumophila*, *M. pneumoniae*, and *M. tuberculosis*. Sample processing and result interpretation were performed according to the manufacturer’s instructions using the RTisochip-W isothermal nucleic acid analyzer (CapitalBiotech Co., Ltd, Beijing, China). According to the manufacturer’s validation data, the assay has a limit of detection of 500 copies/reaction and demonstrated positive and negative agreement rates exceeding 96% and 99%, respectively, compared with sequencing-based reference methods, with an overall agreement rate exceeding 99%. The analytical performance of the assay was established through multicenter clinical validation involving 2,084 sputum specimens collected from five hospitals, and no cross-reactivity with non-target respiratory microorganisms was observed during analytical specificity evaluation. Similar analytical performance of this commercial chip-based LAMP assay has also been reported in independent clinical studies (Kang et al., 2012; Yin et al., 2025).

### 2.5 Standard culture methods

All specimens were subjected to the SCM and identification. Upon arrival at the laboratory, respiratory samples were immediately processed and inoculated by trained microbiology technicians. BALF samples were thoroughly mixed, and at least 5 mL was centrifuged at 3,000 × g for 15 minutes. The supernatant was discarded, and the sediment was used for inoculation. Sputum samples were assessed for quality by Gram staining and microscopic examination. Samples containing >25 neutrophils and <10 squamous epithelial cells per low-power field were considered acceptable for culture. Processed specimens were inoculated onto blood agar, eosin methylene blue (EMB) agar, and chocolate agar plates (Dijing Microbial Technology Co., Ltd, Guangzhou, China), and incubated at 35 °C in a 5% CO_2_ atmosphere (Thermo Fisher Scientific, USA) for 18–24 hours. Suspected colonies were subsequently isolated and subcultured to obtain pure cultures. Species identification was performed using matrix-assisted laser desorption/ionization time-of-flight mass spectrometry (MALDI-TOF MS) (Microflex, Bruker Daltonics, Bremen, Germany).

### 2.6 Statistical analysis

Categorical variables were expressed as frequencies and percentages and compared using Pearson’s chi-square test. A *P*-value of less than 0.05 was considered statistically significant. Agreement between LAMP and SCM was assessed using Cohen’s kappa statistic. According to the criteria proposed by Cohen, Kappa values ≤ 0 indicates no agreement; 0.01-0.20, slight agreement; 0.21-0.40, fair agreement; 0.41-0.60, moderate agreement; 0.61-0.80, substantial agreement; and 0.81-1.00, almost perfect agreement. Statistical analyses were performed using SPSS version 23.0 (IBM Corp., Armonk, NY, USA).

## 3 Results

### 3.1 General clinical data

A total of 5,442 patients were included, with a mean (SD) age of 48 (26.41) years. According to age group classification, children, school-age children, adults, and older adults accounted for 15.99% (870/5,442), 5.71% (311/5,442), 33.46% (1,821/5,442), and 44.84% (2,440/5,442), respectively. Males comprised 59.35% (3,230/5,442) of the cohort, while females accounted for 40.65% (2,212/5,442). Respiratory specimens included BALF and sputum, with BALF accounted for 43.68% (2,377/5,442), as shown in **Table 1**.

**Table 1:** Demographic characteristics and incidence of the included patients from 2020 to 2024.

Patient characteristics	All patients (n=5,442)
Age, n(%)	
0–5 years (Children)	870 (15.99)
6–17 years (School-age children)	311 (5.71)
18–59 years (Adult)	1,821 (33.46)
≥60 years (Older people)	2,440 (44.84)
Gender, n(%)	
Male sex	3,230 (59.35)
Female sex	2,212 (40.65)
Special respiratory specimens, n(%)	
BALF	2,377 (43.68)
Sputum	3,065 (56.32)
Single infection, n(%)	1,424 (26.17)
Multiple infections, n(%)	
Two pathogens	255 (4.69)
Three pathogens	47 (0.86)
Four or more pathogens	8 (0.15)
Overall positive rate, n(%)	1,734 (31.86)

BALF, bronchoalveolar lavage fluid.

### 3.2 Overall infection

**Figure 1:**
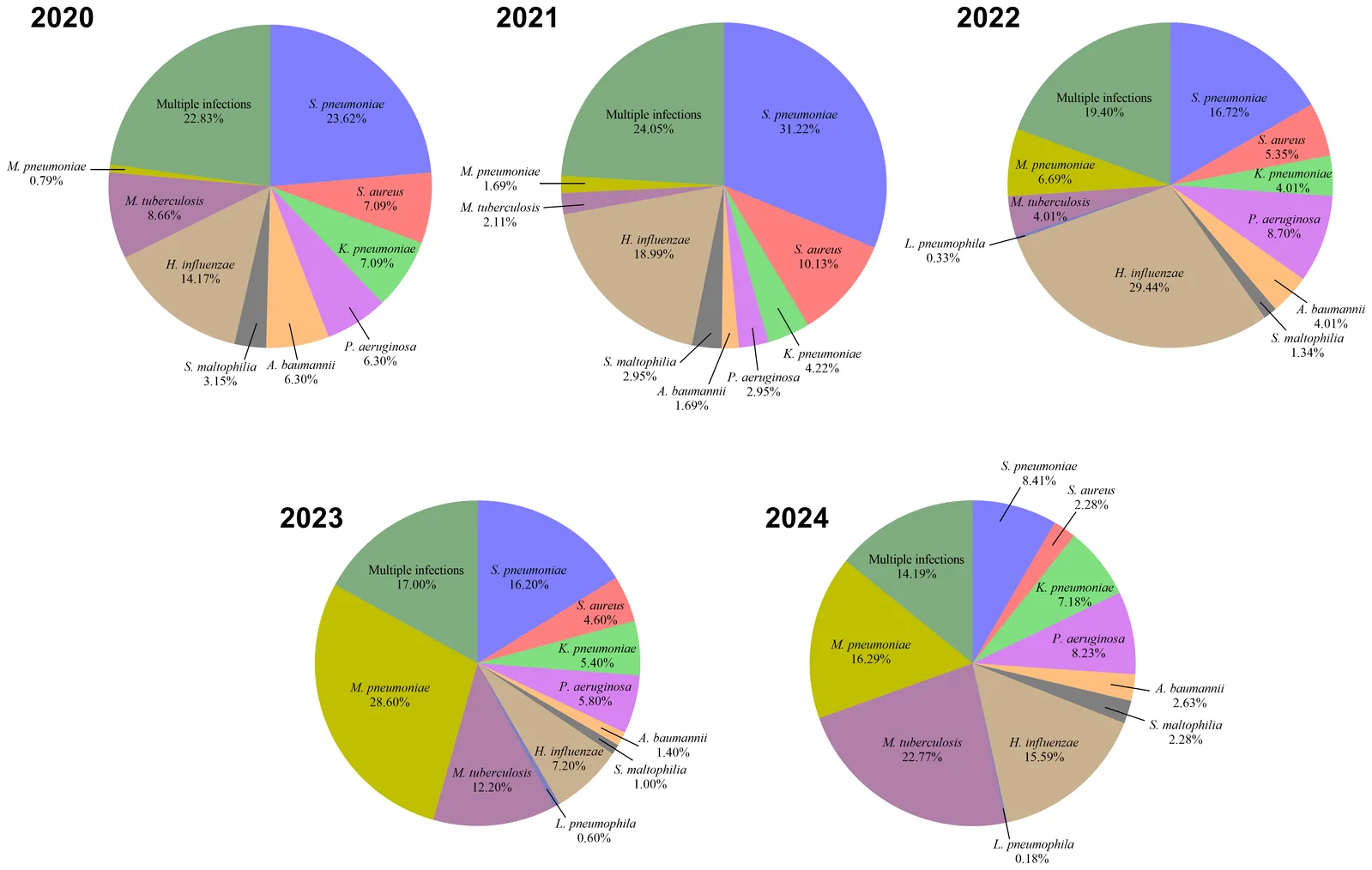
Yearly distribution of respiratory bacterial pathogens detected by LAMP in hospitalized patients with bacterial LRTIs in Jingzhou, from 2020 to 2024. Data are presented separately for each study year.

With the LAMP assay, 1,734 samples tested positive, with an overall positive rate of 31.86% (1,734/5,442). Among these, 1,424 patients (26.17%) were belong to single pathogen infected, 255 (4.69%) two pathogens infected, and 55 (1.01%) three or more pathogens infected. The five most frequently detected pathogens were *H. influenzae* (8.18%, 445/5442), *S. pneumoniae* (7.75%, 422/5,442), *M. pneumoniae* (5.77%, 314/5,442), *M. tuberculosis* (4.56%, 248/5,442), and *K. pneumoniae* (3.27%, 178/5,442). From 2020 to 2024, the positive rates were 30.60% (127/415), 41.80% (237/567), 37.33% (299/801), 34.53% (500/1,448), and 25.83% (571/2211), respectively, showing an overall declining trend from 2021 to 2024. Detailed yearly distributions are presented in **Figure 1**. A stratified analysis according to specimen type was performed to evaluate the potential influence of specimen source on pathogen detection (**Supplementary Table 1**). The overall positive rate was significantly higher in sputum specimens than in BALF specimens (39.05% vs. 22.59%, *P* < 0.001). Significant differences were observed for several pathogens, whereas no significant differences were found for *P. aeruginosa*, *L. pneumophila*, or *M. pneumoniae*.

### 3.3 Seasonal distribution of pathogen detection

**Figure 2:**
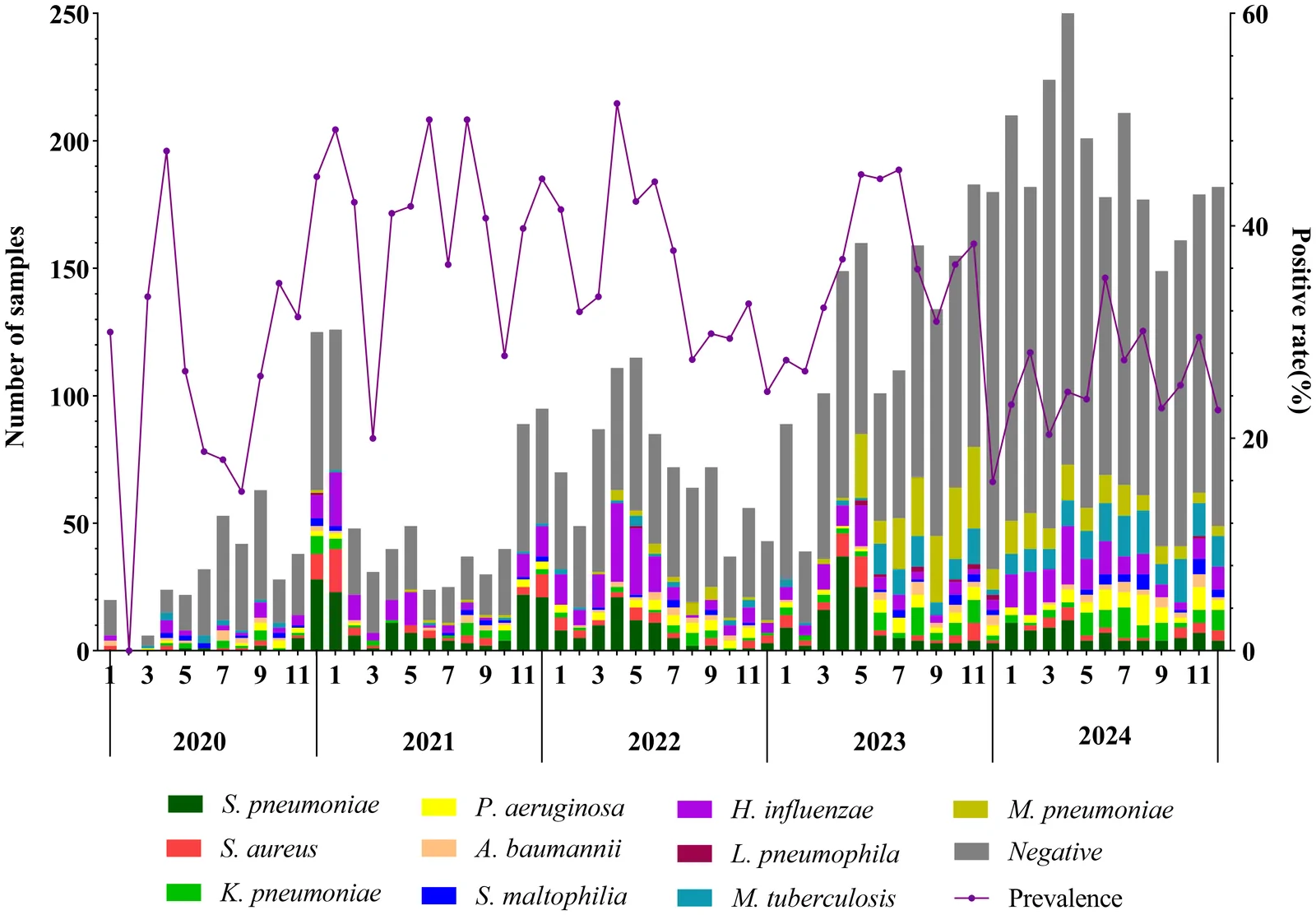
Seasonal trends in the positive rates of respiratory bacterial pathogens in Jingzhou, 2020–2024. The x-axis represents the month of specimen collection. Stacked bars show the numbers of specimens by pathogen detection result (left y-axis), whereas the line shows the overall positive rate (%) (right y-axis). Different colors represent individual pathogens and negative samples.

The overall pathogen positive rate was higher during April to June each year compared to other months. *S. pneumoniae* exhibited a bimodal distribution pattern, with increased positive rates observed in May-June and November-December. The monthly positive rate of *M. pneumoniae* increased markedly between May and November 2023, reaching a peak of 20.16%. In contrast, the monthly distribution of other pathogens appeared relatively scattered without a clear seasonal pattern. Detailed results are shown in **Figure 2**.

### 3.4 Age-specific differences in pathogen spectrum

**Figure 3:**
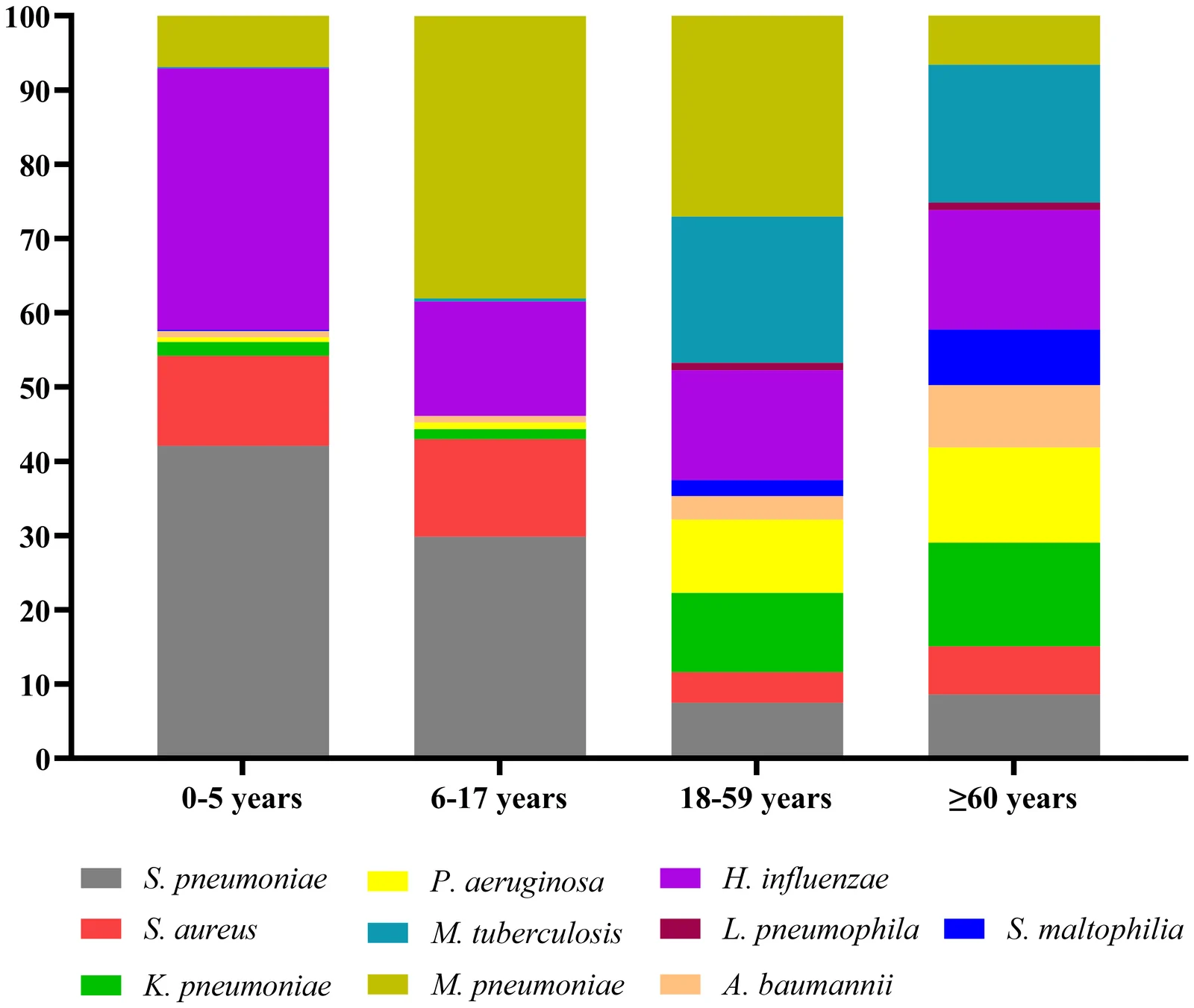
Age-specific distribution of respiratory bacterial pathogens. The x-axis represents the age groups. Stacked bars indicate the percentage distribution of different pathogens within each age group (y-axis, %).

The highest positive rate was observed in school-aged children group (57.88%, 180/311), followed by children group (53.45%, 465/870). The rates were relatively lower in older adults group (26.15%, 638/2,440) and lowest in adults group (24.77%, 451/1,821). Further analysis of pathogen composition demonstrated distinct distribution patterns across age groups. Among children, the four most frequently detected pathogens were *S. pneumoniae* (42.09%), *H. influenzae* (35.19%), *S. aureus* (12.12%), and *M. pneumoniae* (6.90%). The positive rate of *M. pneumoniae* ranked first in school-aged children group, followed by *S. pneumoniae*, *H. influenzae*, and *S. aureus*. In adults and older adults group, *K. pneumoniae*, *P. aeruginosa*, and *M. tuberculosis* were detected at relatively higher frequencies. Detailed results are shown in **Figure 3**.

### 3.5 Variation in pathogen spectrum affected with COVID-19 epidemic

**Figure 4:**
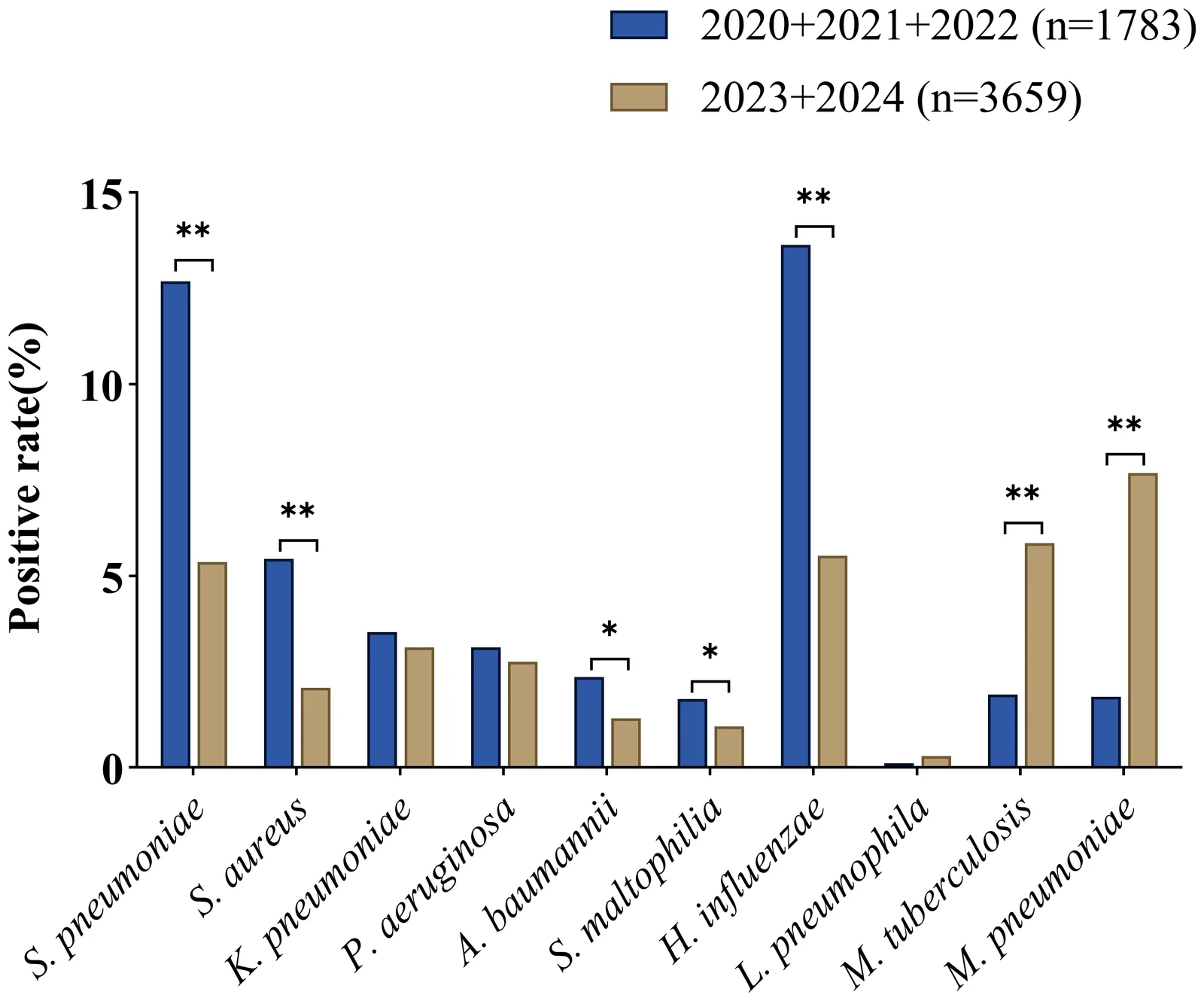
Comparison of pathogen spectrum between the COVID-19 epidemic era (2020-2022) and the post-epidemic era (2023-2024). The x-axis represents the target pathogens, and the y-axis represents the positive rate (%). Bars represent the positive rates during the COVID-19 epidemic era (2020–2022) and the post-epidemic era (2023–2024). Statistical significance is indicated as follows: *P* < 0.05 (*), *P* < 0.01 (**).

During the epidemic era, single-pathogen infections were predominantly caused by *H. influenzae* (13.63%), *S. pneumoniae* (12.68%), and *S. aureus* (5.44%). In the post-epidemic era, *M. pneumoniae* (7.68%), *M. tuberculosis* (5.85%), and *H. influenzae* (5.52%) were the most frequently detected single pathogens. Comparative analysis revealed that the positive rates of *S. pneumoniae*, *S. aureus*, *A. baumannii*, *S. maltophilia*, and *H. influenzae* were significantly higher during the epidemic era than in the post-epidemic era (*P* < 0.05). Conversely, the positive rates of *M. tuberculosis* and *M. pneumoniae* were significantly higher during the post-epidemic era (*P* < 0.001). The comparative results are shown in **Figure 4**.

### 3.6 Analysis of co-infections

**Figure 5:**
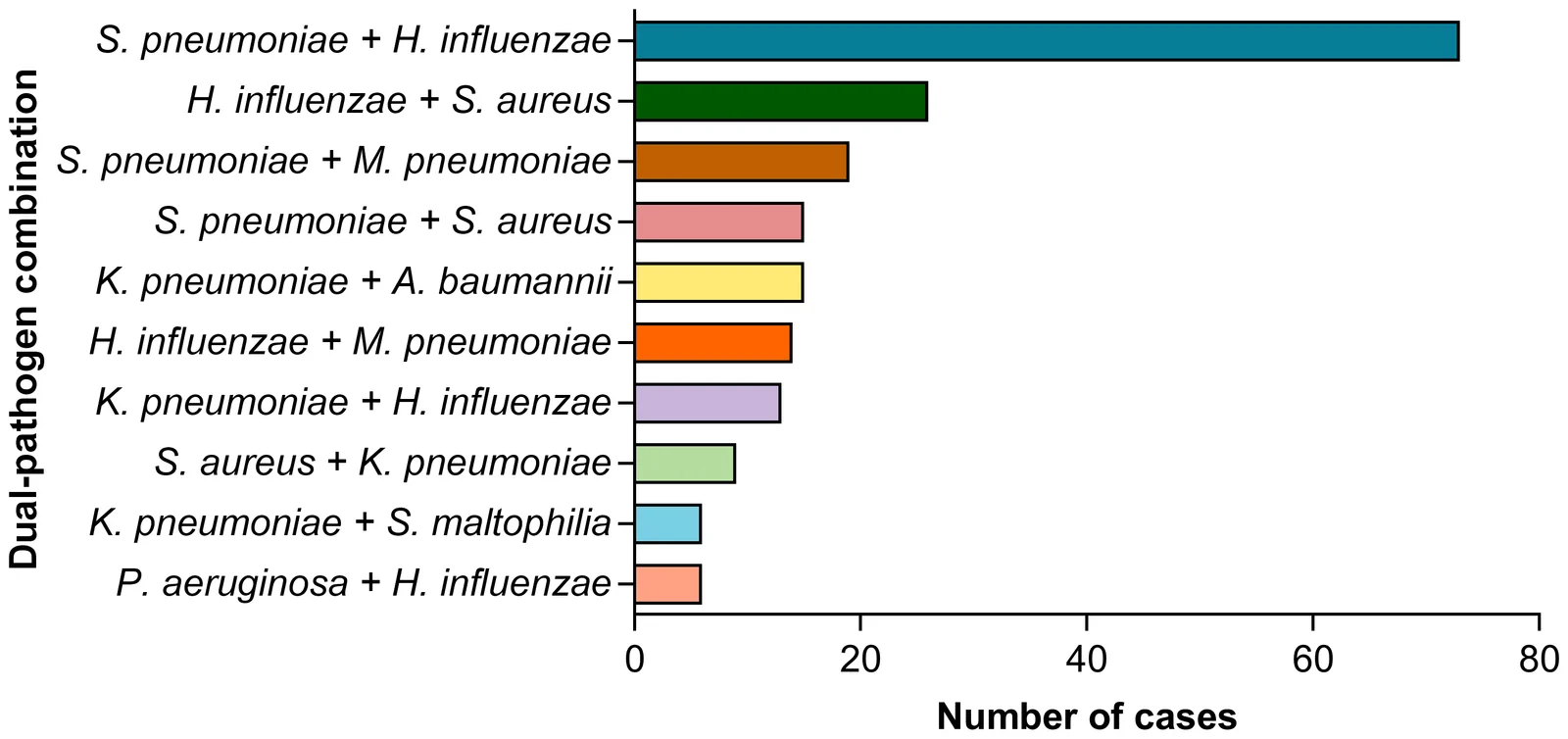
Most common dual-pathogen co-infection combinations detected among patients with bacterial lower respiratory tract infections. Bars represent the number of cases for each dual-pathogen combination.

Among the 1,734 pathogen-positive samples, 310 (17.88%) involved co-infections, including 255 (82.26%) dual-pathogen infections and 55 (17.74%) infections involving three or more pathogens. The co-infection rate differed significantly among age groups (*P* < 0.001), with the highest rates observed in children (13.45%) and school-aged children (11.58%), whereas markedly lower rates were found in adults (2.47%) and older adults (4.59%) (**Table 2**). Co-infection rates also differed significantly by season (*P* = 0.041), as shown in **Supplementary Table 2**. Among dual-pathogen co-infections, the most frequently identified pathogen combination was *S. pneumoniae*–*H. influenzae*, followed by *H. influenzae*–*S. aureus* and *S. pneumoniae*–*M. pneumoniae*, as shown in **Figure 5**. Complete data for all dual-pathogen combinations are provided in **Supplementary Table 3**.

**Table 2:** Age-specific distribution of co-infections.

Age (years)	Total number	No. of co-infections	Prevalence(%)	*P*-value
0-5	870	117	13.45	*χ²* = 158.15 *P* < 0.001
6-17	311	36	11.58	
18-59	1821	45	2.47	
≥60	2440	112	4.59	
Total	5442	310	5.70	

### 3.7 Agreement between LAMP and SCM in BALF samples

A total of 923 BALF samples collected between June 2023 and May 2024 were included to assess the agreement between the LAMP and SCM for respiratory pathogen detection. Among these, 59 samples were positive by both methods, 46 were positive by SCM but negative by LAMP, and 160 were positive by LAMP but negative by SCM. Overall, LAMP showed a higher positive rate than SCM. Agreement results for culturable pathogens are presented in **Table 3**. For *S. aureus*, *K. pneumoniae*, *P. aeruginosa*, *A. baumannii*, *S. maltophilia*, and *M. tuberculosis*, κ values ranged from 0.50 to 0.70, indicating moderate agreement between the two methods. In contrast, poor agreement was observed for *S. pneumoniae* and *H. influenzae*, with LAMP demonstrating higher positive rates than SCM (1.84% vs. 0.33% and 3.58% vs. 0.76%, respectively).

**Table 3:** Comparison of agreement between the LAMP assay and SCM for pathogen detection.

Pathogen	LAMP(+)/SCM(+)	LAMP(-)/SCM(+)	LAMP(-)/SCM(-)	LAMP(+)/SCM(-)	Positive accordance rate	Negative accordance rate	Kappa	*P*-value
*S. pneumoniae*	1	2	904	16	33.33	98.26	0.10	<0.001
*S. aureus*	4	8	911	0	33.33	100.00	0.50	<0.001
*K. pneumoniae*	7	5	904	7	58.33	99.23	0.53	<0.001
*P. aeruginosa*	20	21	880	2	48.78	99.77	0.62	<0.001
*A. baumannii*	2	1	918	2	66.67	99.78	0.57	<0.001
*S. maltophilia*	3	3	916	1	50.00	99.89	0.60	<0.001
*H. influenzae*	3	4	886	30	42.86	96.72	0.14	<0.001
*L. pneumophila*	-	-	920	3	-	99.67	-	-
*M. tuberculosis*	19	2	876	26	90.48	97.12	0.56	<0.001
*M. pneumoniae*	-	-	850	73	-	92.09	-	-

LAMP, loop-mediated isothermal amplification; SCM, standard culture method.

Positive accordance rate = LAMP(+)/SCM(+) ÷ [LAMP(+)/SCM(+) + LAMP(−)/SCM(+)].

Negative accordance rate = LAMP(−)/SCM(−) ÷ [LAMP(−)/SCM(−) + LAMP(+)/SCM(−)].

Agreement analysis was not applicable for *M. pneumoniae* and *L. pneumophila* because these pathogens were not routinely detected by SCM.

## 4 Discussion

This study investigated the epidemiological characteristics of bacterial respiratory pathogens in hospitalized patients with LRTIs in Jingzhou, China, from 2020 to 2024. The overall positive rate was 31.86%, which was higher than those reported in Hong Kong (23.40%) (Liu et al., 2021) and Nepal (17.63%) (Khadka et al., 2022), but lower than those reported in Chongqing (51.32%) (Yin et al., 2025) and Ethiopia (33.50%) (Gebre et al., 2021). The five most frequently detected pathogens were *H. influenzae*, *S. pneumoniae*, *M. pneumoniae*, *M. tuberculosis*, and *K. pneumoniae*, which differed from the pathogen profiles reported in other regions, underscoring the importance of region-specific epidemiological surveillance (Sader et al., 2021). Stratified analysis according to specimen type demonstrated significant differences in the positive rates of several pathogens between BALF and sputum specimens. These differences may reflect variations in specimen origin, contamination or colonization by upper respiratory tract flora, and the clinical indications for specimen collection (Post et al., 2024). Because sputum specimens are more susceptible to contamination by upper respiratory tract microorganisms, they may yield higher positive rates for some pathogens than BALF specimens. Therefore, BALF is generally considered the preferred specimen for reflecting pathogens causing LRTIs. For patients in whom BALF collection is not feasible, the use of qualified sputum specimens, together with appropriate collection procedures such as oral rinsing before expectoration, may help reduce contamination by upper respiratory tract flora. Consequently, specimen type should be taken into account when interpreting pathogen-specific epidemiological findings, particularly in studies including multiple specimen types. Nevertheless, these differences are unlikely to have materially influenced the overall epidemiological patterns observed in the present study.

Seasonal variation analysis further revealed that overall pathogens positive rates were higher between April and June each year, suggesting that the transition from spring to early summer may represent a period of increased respiratory infections in this region. Notably, *S. pneumoniae* exhibited a bimodal distribution pattern, with peaks observed in May-June and November-December. Previous studies have indicated that lower temperatures and increased rainfall may be associated with the colonization and transmission of *S. pneumoniae* in the respiratory tract, which may contribute to the observed seasonal transmission peaks (Domenech de Cellès et al., 2019; Cheliotis et al., 2022). Given this seasonal pattern, implementation of preventive strategies, including targeted vaccination, such as *S. pneumoniae* vaccine, prior to high-risk seasons, may help reduce the associated disease burden in vulnerable populations (Shabil et al., 2024). In addition, the positive rate of *M. pneumoniae* increased markedly between May and November 2023. Similar post-pandemic rebounds of *M. pneumoniae* have also been reported in multiple regions (Zeng et al., 2024; Meyer Sauteur et al., 2025), possibly reflecting changes in population immunity and the relaxation of non-pharmaceutical interventions (Liu et al., 2024). In contrast, the remaining pathogens did not demonstrate clear seasonal patterns, highlighting the need for continuous and systematic regional surveillance.

Distinct age-specific variations were observed in pathogen distribution and positive rates. Positive rates were higher in school-aged children group and children group than in adults group and older adults group, consistent with previous observations that immature immune systems and increased exposure in congregate settings predispose children to respiratory infections (Wei et al., 2024). *S. pneumoniae* and *H. influenzae* predominated among children, consistent with their high nasopharyngeal colonization rates and greater propensity for invasive disease in pediatric populations (Miellet et al., 2024; Zelasko et al., 2024). Among school-aged children, *M. pneumoniae* ranked first, suggesting a shift toward atypical pathogens with increasing age and expanding social interactions (Liu et al., 2023). In contrast, pathogen profiles in adults group and older adults group were more diverse, with higher positive rates of *K. pneumoniae*, *P. aeruginosa*, and *M. tuberculosis*, similar with previous reports (Li et al., 2021). This pattern may reflect the combined effects of a higher burden of comorbidities, age-related immune decline, and repeated healthcare exposure in adults and older adults (Torrance and Haynes, 2022). Although the overall positive rates were relatively lower in these groups, the pathogen spectrum was more diverse, with a higher prevalence of organisms associated with potential antimicrobial resistance. Such a distribution may predispose these patients to an increased risk of severe infections and adverse clinical outcomes, warranting particular attention in the context of population aging (Häder et al., 2023; Chu et al., 2024). Taken together, age-specific differences should be considered when formulating empirical antimicrobial strategies and stewardship policies to enable more precise risk stratification and targeted management.

Single-pathogen infections predominated in this study, consistent with previous reports from Hanzhong (Zheng et al., 2025) and Nigeria (Uzoamaka et al., 2017). This predominance may reflect ecological competition among bacterial species within the host, whereby pathogens with competitive advantages are more likely to predominate (Hibbing et al., 2010). Nevertheless, polymicrobial infections accounted for nearly one-fifth of all pathogen-positive cases and should not be overlooked, particularly in immunocompromised patients and those with ventilator-associated pneumonia (Kreitmann et al., 2024). Among dual-pathogen co-infections, the combination of *S. pneumoniae* and *H. influenzae* was the most frequently identified. These two organisms commonly colonize the upper respiratory tract and have been reported to coexist through complex ecological interactions, which may facilitate concurrent infections under certain clinical conditions (Tanaka et al., 2026). Co-infections may also complicate empirical antimicrobial therapy because different pathogens often exhibit distinct antimicrobial susceptibility profiles (Liu et al., 2023). Co-infections were more common among children and school-aged children than among adults, possibly owing to immature immune function and increased exposure to respiratory pathogens in childcare and school settings (Lloyd and Saglani, 2023).

Distinct differences in pathogen spectrum were observed between the COVID-19 epidemic era (2020-2022) and the post-epidemic era (2023-2024). During the COVID-19 epidemic era, *S. pneumoniae*, *S. aureus*, and *H. influenzae* predominated, while hospital-associated pathogens, including *A. baumannii* and *S. maltophilia* also exhibited higher positive rates compared with the post-epidemic era. These finding may be associated withe the relatively greater disease severity among hospitalized patients, increased use of invasive procedures, and a higher risk of healthcare-associated infections during the pandemic (Brooke, 2021; Zhang et al., 2022). In addition, non-pharmaceutical interventions implemented during the COVID-19 pandemic may have altered the transmission dynamics and ecological balance of respiratory pathogens (Li et al., 2022; Kajihara et al., 2024). In the post-epidemic era, positive rates of *M. pneumoniae* and *M. tuberculosis* increased, suggesting a gradual restoration of community transmission patterns. Although several studies have reported a rebound in respiratory pathogen positivity following the relaxation of control measures (Gilca et al., 2024; Li et al., 2024), the overall positive rates in Jingzhou showed a declining trend from 2021 to 2024 and remained relatively low (25.83%). This discrepancy may be attributable to increased healthcare-seeking behavior among patients with mild symptoms after the pandemic, resulting in a larger proportion of less severe cases being included. Consequently, the overall positive rate may have been diluted at the population level. Continuous and dynamic surveillance is warranted to better characterize long-term epidemiological trends.

The present study demonstrated that LAMP exhibited a higher positive rate than SCM, consistent with previous reports that nucleic acid-based assays are more sensitive than conventional culture methods (Si et al., 2021). Moderate agreement was observed for several pathogens (*S. aureus*, *K. pneumoniae*, *P. aeruginosa*, *A. baumannii*, *S. maltophilia*, and *M. tuberculosis*), whereas poor agreement was found for *S. pneumoniae* and *H. influenzae.* This discrepancy may be explained by the inherent limitations of culture-based methods, which depend on growth conditions, operator expertise, and the ability to distinguish pathogens from commensal flora in complex respiratory samples (DeYoung et al., 2022). In contrast, nucleic acid-based assays can directly detect pathogen-specific genetic material, including low-abundance targets, thereby improving detection sensitivity. As a rapid and efficient molecular technique, LAMP enables simultaneous detection of multiple pathogens within one hour, with relatively simple operational and interpretation (Garg et al., 2022). With ongoing advances in molecular diagnostics, its integration with other platforms may further enhance its clinical utility (Wu et al., 2022; Shi et al., 2025). Overall, these findings suggest that the LAMP assay may serve as a useful complementary diagnostic tool for the rapid detection of respiratory bacterial pathogens. Its potential application in primary healthcare settings warrants further evaluation in prospective clinical studies.

This study has several limitations. First, the commercial LAMP assay used in this study is a targeted molecular assay designed to detect only predefined respiratory pathogens. Consequently, mixed infections involving respiratory viruses, fungal pathogens, or other non-target microorganisms could not be comprehensively evaluated. Second, detailed clinical information was not included in the present analysis. Therefore, associations between pathogen profiles and disease severity or clinical outcomes could not be assessed. Third, discrepant results between the LAMP assay and SCM were not further validated using independent molecular methods, such as qPCR or next-generation sequencing (NGS), which may have affected the interpretation of certain discordant findings. Finally, as a single-center study conducted in Jingzhou, the generalizability of our findings to other regions may be limited. Future multicenter studies incorporating broader pathogen panels, comprehensive clinical data, and multi-method validation strategies are warranted to provide a more comprehensive understanding of the epidemiology of bacterial LRTIs.

## 5 Conclusion

This study systematically characterized the epidemiological features of bacterial LRTIs in Jingzhou, China, and identified distinct variations in pathogen distribution across age groups, seasons, and different phases of the COVID-19 pandemic. These findings provide important epidemiological evidence to support empirical antibacterial therapy and age-stratified prevention and control strategies for bacterial LRTIs. Given its operational simplicity and relatively low equipment requirements, the LAMP assay may represent a promising molecular diagnostic tool for rapid detection of common bacterial respiratory pathogens. However, its potential applicability in primary healthcare settings warrants further evaluation in prospective studies.

## Data Availability

The raw data supporting the conclusions of this article will be made available by the authors, without undue reservation.
