# Supplementary material for: Epidemiological characteristics of bacterial lower respiratory tract infections in Jingzhou, Central China, 2020-2024
Source: Front Cell Infect Microbiol. 2026 Aug 26;16:1869915. doi: 10.3389/fcimb.2026.1869915 (PMC13553388; doi:10.3389/fcimb.2026.1869915)
Supplement: Supplementary file 1 [file Table1.docx]

**Supplementary Table S1.** Comparison of pathogen positivity rates between bronchoalveolar lavage fluid (BALF) and sputum specimens.

| Pathogen | BALF (n=2,377) | Sputum (n=3,065) | *χ²* | *P*-value |
| --- | --- | --- | --- | --- |
| *S. pneumoniae* | 47 (1.98%) | 375 (12.23%) | 196.92 | <0.0001 |
| *S. aureus* | 17 (0.72%) | 156 (5.09%) | 83.24 | <0.0001 |
| *K. pneumoniae* | 50 (2.10%) | 128 (4.18%) | 18.18 | <0.0001 |
| *P. aeruginosa* | 72 (3.03%) | 85 (2.77%) | 0.31 | 0.5761 |
| *A. baumannii* | 17 (0.72%) | 72 (2.35%) | 22.22 | <0.0001 |
| *S. maltophilia* | 13 (0.55%) | 58 (1.89%) | 18.82 | <0.0001 |
| *H. influenzae* | 80 (3.37%) | 365 (11.91%) | 130.13 | <0.0001 |
| *L. pneumophila* | 5 (0.21%) | 8 (0.26%) | 0.14 | 0.7042 |
| *M. tuberculosis* | 149 (6.27%) | 99 (3.23%) | 28.42 | <0.0001 |
| *M. pneumoniae* | 137 (5.76%) | 177 (5.77%) | 0.0003 | 0.9858 |
| Total | 537 (22.59%) | 1,197 (39.05%) | 167.11 | <0.0001 |
