# Supplementary material for: Epidemiological characteristics of bacterial lower respiratory tract infections in Jingzhou, Central China, 2020-2024
Source: Front Cell Infect Microbiol. 2026 Aug 26;16:1869915. doi: 10.3389/fcimb.2026.1869915 (PMC13553388; doi:10.3389/fcimb.2026.1869915)
Supplement: Supplementary file 2 [file Table2.docx]

**Supplementary Table S2. Seasonal distribution of co-infections**

| Seasons | Total number | No. of co-infections | Prevalence(%) | *P*-value |
| --- | --- | --- | --- | --- |
| Spring | 1462 | 104 | 7.11 | *χ²*=8.25  *P*=0.041 |
| Summer | 1283 | 67 | 5.22 |  |
| Autumn | 1905 | 103 | 5.41 |  |
| Winter | 792 | 36 | 4.55 |  |
| Total | 5442 | 310 | 5.70 |  |
