# Supplementary material for: Epidemiological characteristics of bacterial lower respiratory tract infections in Jingzhou, Central China, 2020-2024
Source: Front Cell Infect Microbiol. 2026 Aug 26;16:1869915. doi: 10.3389/fcimb.2026.1869915 (PMC13553388; doi:10.3389/fcimb.2026.1869915)
Supplement: Supplementary file 3 [file Table3.docx]

**Supplementary Table S3.** Distribution of pathogen combinations in dual-pathogen co-infections.

| Pathogen | *S. pneumoniae* | *S. aureus* | *K. pneumoniae* | *P. aeruginosa* | *A. baumannii* | *S. maltophilia* | *H. influenzae* | *L. pneumophila* | *M. tuberculosis* | *M. pneumoniae* |
| --- | --- | --- | --- | --- | --- | --- | --- | --- | --- | --- |
| *S. pneumoniae* | 118 | 15 | 3 | 1 | 4 | 1 | 73 | 0 | 2 | 19 |
| *S. aureus* | 15 | 60 | 9 | 2 | 2 | 2 | 26 | 0 | 3 | 1 |
| *K. pneumoniae* | 3 | 9 | 52 | 4 | 15 | 6 | 13 | 0 | 2 | 0 |
| *P. aeruginosa* | 1 | 2 | 4 | 24 | 1 | 5 | 6 | 0 | 5 | 0 |
| *A. baumannii* | 4 | 2 | 15 | 1 | 31 | 3 | 3 | 0 | 2 | 1 |
| *S. maltophilia* | 1 | 2 | 6 | 5 | 3 | 18 | 0 | 0 | 1 | 0 |
| *H. influenzae* | 73 | 26 | 13 | 6 | 3 | 0 | 139 | 0 | 4 | 14 |
| *L. pneumophila* | 0 | 0 | 0 | 0 | 0 | 0 | 0 | 3 | 1 | 2 |
| *M. tuberculosis* | 2 | 3 | 2 | 5 | 2 | 1 | 4 | 1 | 24 | 4 |
| *M. pneumoniae* | 19 | 1 | 0 | 0 | 1 | 0 | 14 | 2 | 4 | 41 |

Notes: Only dual-pathogen co-infections (n = 255) were included in this analysis. Cases with three or more pathogens (n = 55) were excluded because of their low frequency and heterogeneous pathogen combinations.
